# Towards origins of virtual artificial life: an overview

**DOI:** 10.1098/rstb.2024.0298

**Published:** 2025-10-02

**Authors:** Susan Stepney

**Affiliations:** ^1^ Department of Computer Science, University of York, York YO10 5DD, UK

**Keywords:** artificial life, autopoiesis, agency, open-endedness, adaptation, embodiment

## Abstract

The field of artificial life (ALife) studies ‘life as it could be’, in contrast to biology’s study of ‘life as we know it to be’. This includes a wide range of potential physical substrates, from synthetic biology (new genes), through xenobiology (new amino acids and DNA bases), inorganic chemistry (different structural elements), soft and hard robotics (new kinds of bodies) and also virtual life (existing inside a computer). Since any such life forms are artificial, the originating mechanisms can be similarly artificial, or can attempt to emulate natural mechanisms. Given the wide range of possible substrates and origins, it is crucial to have good definitions, and well-defined ways to detect and measure life, if and when it originates. This overview examines the current state of the art in ALife in defining, detecting and originating its subject matter, with its main focus on virtual life. After discussing common properties of several definitions of life, the overview synthesizes an engineering-focussed definition, in terms of abstract requirements, generic designs and specific implementation mechanisms, and then reviews the current state of the art through this lens. Although virtual ALife that satisfies all these requirements is yet to be exhibited, significant progress has been made on engineering individual mechanisms and, arguably, partially alive systems.

This article is part of the theme issue ‘Origins of life: the possible and the actual’.

## 1. Introduction

Biology is the study of terrestrial life, *life as we know it*. It is not necessary to know how this life originated in order to study it, but this major transition [1] is itself a core intellectual question. The origin of life question is, how did brute non-living matter come to form the beginnings of the complex, evolved web of life we see on Earth today? This question is relevant to the search for extra-terrestrial life: what are the conditions needed for its origin? Do these have to adhere closely to original terrestrial conditions, or can they vary, originating wildly different kinds of life? It is also relevant to the broader question: what is the range of possibilities for life itself to originate and exist?

Study of the origin of terrestrial life is made difficult not only by the fact that the relevant processes happened billions of years ago in an environment very different from today’s, but also because subsequent evolution may have wiped out evidence of early transitional stages: initial terrestrial life may have been very different from the life we see today. Furthermore, it is difficult to distinguish necessary from contingent properties given only one example: ‘replaying the tape’ [2] might result in very different lifeforms.

The overall goal of the interdisciplinary research domain called artificial life (ALife) is to understand the science and engineering of *life as it could be* in general [3], including physico-chemical systems, physico-mechanical systems and virtual systems. It provides an alternative route for a more general study of the origins of all forms of life through laboratory experiments, simulations and theoretical approaches, drawing on and influencing fields as diverse as computational biology, synthetic biology, biochemistry, inorganic chemistry, soft and hard robotics, complexity and computational simulation. This alternative study is also difficult: there is no consensus regarding what constitutes life, so how do we know when we have achieved it? And there are as yet no credible examples of life artificially produced from non-living starting points, so there is no actual subject matter to study. Nevertheless, research in ALife continues.

This overview covers the current state of the art of research into the origins of virtual ALife and what insights it might yield into the origin of terrestrial life, and the wider concept of life generally.

## 2. Defining life

Discussions of the origins of (artificial) life would be helped by having some definition of life itself, in order to clarify just what is originating, or being originated.

There is a large biological literature on defining life, its subject matter. This needs to be considered carefully when tackling ALife, where artifice may replace some of the supposed necessary processes or properties of life. Rather than going into these definitions in depth, here we just highlight some of the commonly identified processes and components, and evaluate them in the context of general ALife.

### (a) Life, living, alive

Benner [4] points out that it is necessary to be precise in one’s definitions, in particular distinguishing the system-level concept of ‘life’ and the individual-level property of ‘being alive’. Conflating the two can lead to many difficulties; for example, requiring the characteristic of ‘being able to reproduce’ leads to complaints that individual animals are therefore not alive because they cannot reproduce individually: it takes two. Researchers typically investigate individual living beings, or small ecosystems, rather than planetary-scale life itself.

Here, we use the following terminology:

— *living* (adj.): the process, in contrast to non-living; ‘the living state may best be assessed at an ecosystem or planetary scale’ [5]
  — *alive*: a being or system that is currently living
  — *capable of living*: not currently performing any living processes, but able to become alive with a change of situation (e.g. spores, dehydrated cells) [6, p.278]
  — *dead*: previously alive, but no longer so, and not capable of living
— *non-living* (adj.): neither alive, nor capable of living nor dead
— *life* (noun): a whole system, ecosystem or biosphere, of living beings

### (b) Kinds of life

It is useful to break kinds of life into categories (box 1), as the form of the origin question can be different in different cases.

Box 1. **A classification of various existing and potential forms of life. Some instances might incorporate several classes, for example: a combination of hard robotic body with neural brain, of mechanical and chemical substrates.**

(i) natural life
  (a) terrestrial life (biology)
  (b) extraterrestrial life (exobiology)
(ii) artificial life
  (a) top-down construction
    (i) somatic (body modification)
      (A) drugs, surgery (medical intervention)
      (B) tissue engineering
      (C) cyborgs (prosthetics, augments)
    (ii) genetic
      (A) selective breeding (agriculture)
      (B) genetic engineering (systems biology)
      (C) xenobiology
  (b) bottom-up construction
    (i) physico-chemical substrate (protocells; organic and inorganic chemistry)
    (ii) physico-mechanical substrate (soft/hard/mixed robots)
    (iii) computational substrate (virtual life)

Natural life, which is life that has come into being with no intervention from pre-existing life forms, can be categorized as: (i) terrestrial life—life as we know it on Earth—the subject matter of Biology; and (ii) extraterrestrial life—life as it might exist elsewhere and that has originated independently of terrestrial life—the subject matter of Exobiology.

ALife, here meaning life where human^1^ artifice is involved in its construction, can be categorized as: (i) top-down, starting with a living system and modifying it; either somatic (modifying at the organism or tissue level, through chemical or physical means), or genetic (modifying the genome, through artificial selection or genetic engineering, or even adding new components to the genome: xenobiology); and (ii) bottom-up, starting with non-living materials and persuading them to come to life.

### (c) Existing definitions

#### (i) Biology

Terrestrial biology is the study of *life as we know it*. It is generally agreed that living systems are not comprised of any kind of material or energy that is unique to them: they are made of the same underlying stuff as non-living systems, albeit organized differently.

Although most biologists leave aside attempts to define the subject matter of their discipline, there have been multiple attempts to define it. Trifonov [8] identifies and analyses 123 different and often conflicting definitions, and extracts a minimal definition from them: ‘Life is self-reproduction with variations’, which takes only an evolutionary view. Biological definitions may focus on specifics of *terrestrial* life, whereas a more general definition could encompass other forms. One such general definition comes from Harold [9, p. 232], who notes that there are two main camps in the definition of life; one focusses on ‘autopoietic systems: self-constructing, self-maintaining, energy transducing autocatalytic entities’, the other on Darwinian evolution (as in the Trifonov definition above). Harold combines them into: ‘life is the property of autopoietic systems capable of evolving by variation and natural selection’.

#### (ii) Exobiology

Exobiology, also called astrobiology, is the study of putative (natural) extraterrestrial life. Research focusses mainly on search for its existence; its origins would be the next step. This subject requires a broader definition of life that is not predicated on necessarily being the same, particularly in material composition, as terrestrial life. Such broader definitions do tend to be inspired by terrestrial life as we know it, but may encompass ALife, too. Bartlett & Wong [5] discuss their broader definition. In order to distinguish it from specifically terrestrial life, they coin a new term for this broader case: lyfe (pronounced ‘loif’). They define four processes necessary for full lyfe: dissipation of free energy, autocatalysis, homoeostasis and learning.

Life exists in a context: an environment that provides resources, accepts waste and interacts with and is affected by the living system in complex ways. Wong *et al.* [10] emphasize the need for considering the context that supports and constrains particular forms of life. In particular, they note that *biosignatures*—observables that are evidence for the existence of life, now or in the past, but are not necessarily themselves alive—are context- or environment-dependent: the production of a terrestrial biosignature in a very different environment is not necessarily a sign of life in that alien environment, because it would be so different that it would produce different biomarkers. Hence terrestrial biosignatures may not be appropriate markers for ALife either. The most generic biomarkers are far-from-equilibrium conditions [11], such as skewed isotopic, molecular, sedimentary or morphological distributions; even here, however, complex pre-biotic geo-chemical processes can alter systems in surprisingly complex ways [12].

#### (iii) Issues with existing definitions

Many of the existing definitions of life, even those designed to incorporate possible exobiologies, are to some degree problematic, being what Benner [4] refers to as ‘list definitions’: a list of allegedly necessary and sufficient properties and processes to separate the living from the non-living. Many such properties are phenotypic, discernable from the form or functioning of a living organism (metabolism, compartments); others are at a population or system level (Darwinian evolution). Maynard Smith & Szathmáry [1, p. 17] point out the issues with relying on phenotypic definitions: many clearly non-living systems can share phenotypic properties with living systems.

Cleland & Chyba [13] argue we need a theory before we can make definitions. Any such theory needs to conform to more fundamental physical theories, in particular, thermodynamics. Consider the oft-quoted NASA definition: ‘*life* is a self-sustained chemical system capable of undergoing Darwinian evolution’ [14]. Benner [4] argues that the NASA definition constitutes what he calls a *definition-theory*: a theory implicit in, or implied by, a given definition. The NASA theory constrains what astrobiologists look for: in particular, chemical metabolic networks that support Darwinian evolution. Benner argues that a more generic definition of *metabolism* as the ‘ability to exploit free energy to transform matter “without” into matter “within”’ is universal, in that ‘our view of life would need to change dramatically were we to include life without metabolism’. He also says that the NASA definition is relevant only to natural life, not ALife, because explicitly engineered ALife need not undergo Darwinian evolution, even though the natural lifeforms that engineered it have done so.

ALife authors typically attempt abstractions that divorce their definitions from known substrates. For example, Adami takes an information-theoretic thermodynamic approach, to provide what he calls a *principle* of living systems:

Life is a property of an ensemble of units that share information coded in a physical substrate and which, in the presence of noise, manages to keep its entropy significantly lower than the maximal entropy of the ensemble, on timescales exceeding the ‘natural’ timescale of *decay* of the (information-bearing) substrate by many orders of magnitude. [15, p. 6]

## 3. An engineering definition

One major issue with current definitions is that they exist at different levels, and may even combine aspects of different levels. For example, some properties are at a population level (reproducing, undergoing Darwinian evolution) while others are at an organism level (having a metabolism). They are also a mix of low-level *mechanisms* (e.g. metabolism, reproduction, evolution) and high-level *properties* (e.g. autopoiesis, adaptation). For example, Harold’s [9, p. 232] definition quoted earlier combines top-down autopoiesis and bottom-up Darwinian evolution.

ALife is by definition *artificial*: there is an artificer, or engineer, involved in its production and origin. This makes it reasonable to use an engineering approach to the definition, which helps to separate out some categories and resolve some of the issues with defining any form of life, natural or artificial. This engineering approach encourages us to define three levels of life properties: requirements, design and implementation.

### (a) Requirements

The 'requirements’ level covers the abstract concepts that are considered to be the defining *properties* of life, without consideration of specific mechanisms or implementation concerns. Any implemented system that meets these requirements is deemed to be living. Here, we take these concepts to be: autopoiesis, agency and open-ended adaptation.

*Autopoiesis*. One of the key distinguishing features of a living being is that it creates and maintains itself (albeit exploiting external resources, material and energy, to do so). *Autopoiesis*, the concept introduced by Varela and coworkers [16,17], is this self-referential property of a being continually producing and re-producing itself.^2^ So a living being (or system) maintains its identity over time, even as its parts may change.

*Agency*. A living being is an active *being*, rather than a mere reactive brute *thing*. A living being has agency: the ability to manipulate itself and its environment to achieve *autonomously* determined goals through purposive functional *behaviours*, the least of which are autopoietic self-construction and self-preservation, and possibly reproduction, although not all living beings need reproduce. The material parts of a living being (e.g. cells and tissues) also arguably have agency [18].

*Open-ended adaptation*. Living beings exist in a context of, and interact with, other living beings and a non-living dynamic open environment. In order for them to survive and thrive, they need to autonomously *adapt* to this external context. Other beings will also be adapting, and the environment will be changing, owing both to external factors and to its interactions with life, so this needs to be a continual process of adaptation. It should also be *open-ended*, in that the adaptive changes can demonstrate continual novelty as they respond to an open, changing environment.

*Summary of requirements*. Given this, we can define the *requirements* for living beings and living systems as follows. A ‘living being’ is an *autopoietic* system that has agency; it *interacts* with its *environment* in the pursuit of its *goals* through *purposeful behaviour*; it *adapts* to changing circumstances. A ‘living system’ is a *collection* of living beings *interacting* and open-endedly *co-adapting* with the other living beings and a non-living *environment*. We use these requirements to outline a design for living beings that fulfils them (§3c).

These requirements are for ‘full’ life; for a living system at least as rich and complex as the terrestrial biosphere. However, systems only partially fulfilling such requirements may also be considered to be living to a lesser or greater degree. Bartlett & Wong [5] allow the possibility of sub-lyfe, exhibiting only a subset of the required processes (§2c(ii)). Other authors also allow gradations of life; in particular, Benner [4] allows for ALife forms that cannot self-maintain, but require continual input from their makers; Rasmussen *et al.* [19, p. xiii] defined a minimal physico-chemical living system that exhibits a degree of autopoiesis and evolution.

### (b) Virtual life

Existing definitions of life, even those broadened to include possible exobiological definitions, typically assume physical, material embodiment. What of virtual life, realized in a (necessarily physical) computational medium? It is reasonable to assume that any form of virtual life is *artificial*, since the medium is artificial; however, the question remains: is *virtual* life possible?^3^

The requirements listed in §3*a* do not seem to require physical instantiation (other than the underlying physical system supporting the computational processes). Rosen [21], however, argues that virtual (software) life is not possible. He defines a living organism thus [21, p. 244]:

*a material system is an organism if, and only if, it is closed to efficient causation.*^4^ That is, if **f** is any component of such a system, the question ‘why **f**?' has an answer within the system, which corresponds to the category of efficient cause of **f**.

According to Rosen, this closure property makes an organism distinct from a *machine*. In machines, there is a ‘characteristic absence of closed chains of efficient causation’ [21, p. 246], that is, when asking the question ‘why **f**’, ‘any such answer must pertain to the *environment* of the system’ [21, p. 246]: the efficient cause of **f** is in the environment, not the system itself. He also argues that software running on hardware is a machine [21, ch. 9]. Hence an executing program is a machine, and therefore not an organism, not alive, not virtual life. Rosen’s definition has striking similarities to that of autopoiesis, but he reaches the opposite conclusion; the original definition of autopoiesis (see §4*b*) uses a mathematical model, a virtual system, a *machine* in Rosen’s terms. Rosen’s work is notoriously opaque, and has spawned much literature attempting to unpick it and elaborate on it, with authors coming to different conclusions about virtual life [22–24].

A related concept is that of Pattee’s self-referential relation of *semantic closure* [25], where ‘only by virtue of the freely selected symbolic aspects of matter do the law-determined physical aspects of matter become functional’ [26]. Danchin [27] makes a similar point that it is *symbolic relationships* between parts, not their detailed physico-chemical properties, that are important, and those relationships can be arbitrary—for example, the genetic code; since the relationships could be different, they are not reducible to physics. In a sense, the crucial symbolic, informational level of life is like a *virtual machine* atop some underlying substrate. Emergent properties are in some sense independent of their substrate: different substrates might exhibit similar emergent properties [28]. This argument implies that the required closure can be implemented in software; a form of semantic closure has been demonstrated in an automata chemistry experiment [29].

The argument still rages. For the purpose of this article, we assume that virtual life is possible.

### (c) Design level

The design level defines the *generic* mechanisms or processes that operationalise, or realize, the requirements. A given mechanism may realize only part of a requirement, and may realize parts of different requirements. Some of these mechanisms explicitly realize a requirement, others realize more implicit consequences of the requirements. The mechanisms outlined here are generic and high-level; nevertheless, there may be other designs that realize the requirements differently.

Self-* processes. Autopoietic systems are self-contained. They are ‘closed in production’, in that their components enable processes that give rise to those components; they are ‘closed in space’ in that they construct their own boundary between their self and the surrounding environment [30]. They can be designed with the so-called self-* processes of self-producing, self-assembling, self-creating. Autocatalysis is a key concept in self-sustaining self-production; Kaufmann demonstrated how readily autocatalytic sets of interacting components could be produced [31]. Design also needs to include self-maintaining, self-regulating, self-sustaining, self-preserving, self-repairing, self-healing processes, owing to errors during self-production and to perturbative interactions with a potentially damaging environment. These processes cover *homoeostasis*—maintaining or returning to a stable internal state and keeping state values within a certain range, despite perturbations and fluctuations, which is a process of dynamic stability that requires feedback—and *homoeorhesis*—maintaining or returning to a stable trajectory (e.g. of desired change, growth), despite perturbations and fluctuations.

*Embodiment*. The need to build the self out of environmental material, and interactions with and adaptation to the environment, are often addressed through *embodiment*, of being situated in and interacting with a complex environment. Embodiment covers both mind, through embodied cognition [32], and body, through physical dynamics [33,34] giving embodied intelligence [35].

The complex environment becomes a direct resource for the embodied system. Thermodynamically, a physical living system is *open*^5^ to environmental inputs that are the source of the material needed for autopoietic self-construction and of the free energy needed for behaviour; it is dissipative, and the embodying environment is the sink for its resulting entropic waste products. To avoid degradation to equilibrium (death), the living system needs to self-maintain far-from-equilibrium on a free energy gradient to continue to act. Stochastic thermodynamics may be a necessary tool to study the origin of small chemical systems, and how they can deviate from thermodynamic equilibrium [36].

Complex feedbacks between the embodied self and embodying environment also allow offloading of computational and cognitive effort to the environment, for example, by exploiting the dynamics of the body to support behaviours [34] and by using ‘the world as its own model’ [37].

This embodied situatedness and interaction are typically assumed to be with respect to a *physical* environment, but could potentially be with respect to a virtual environment: ‘Embodiment can be viewed as a property not just of situated material systems, but of any suitably complex system engaged in a complex intertwined feedback relationship with its suitably complex environment’ [38].

*Information and computation*. Adaptation requires some form of state that can change in response to an experience, so that a different action can be taken in response to the same stimulus to produce a different, hopefully better adapted, outcome. These properties are related to information storage, or *memory*. Interaction with, and response to, an external environment implies the existence of *sensors* to determine the state of the environment and self, and of *actuators* to alter the state of the environment and self. Choice of behaviour to achieve a goal requires some form of information processing, or computation. So the requirements of adaptation, interaction and goal-driven behaviour all point to the need for a *cognitive* system that can perform both information storage and processing, coupled with sensors and actuators.

*Evolution*. Darwinian evolution by *inheritance* with *variation* and *selection* is a mechanism of maintenance and adaptation across generations, hence using a broad definition of ‘self’. Inheritance comes from *reproduction*, the production of an offspring that is like the parent self/selves in some manner. Variation comes from imperfect *replication*: replication is the copying of the *genome*, the information content needed to produce a new self or new part of a higher-level self (e.g. cell division); imperfect replication may help support adaptation by producing a variant self. Selection comes from differential rates of survival in a complex environment.

Darwinian evolution is the ground floor of Dennett’s Tower of Generate-and-Test [39, ch. 13.I] framework for adaptation.^6^ As beings complexify, they can move up the tower and exploit more sophisticated mechanisms that allow them to adapt during their individual lifetimes, including reinforcement learning, mental modelling, social learning and tool use.

*Complexity*. The need for increasing complexity is a consequence of the requirement for *open-ended* adaptation. The continual production of new, previously unseen behaviours needs new, previously unseen structures to support them. The system under design needs to incorporate this continual novelty, typically structured into hierarchies to allow for modularity, reuse and more compact representations in the related information content.

### (d) Implementation level

The implementation level defines *specific* mechanisms that together realize a design. There may be alternative implementations that realize a design differently. There are many mechanisms suggested and examined. *Life as we know it*, biology, provides a base set of mechanisms. *Life as it could be*—ALife—examines variants or alternatives to these, to understand better what is necessary, and what is merely contingent, about natural life; some of these mechanisms are discussed in section 4.

Actual engineering of ALife naturally focusses on the implementation level, as this is what produces concrete realizations. Owing to the scale and complexity of the endeavour, much of the research focusses on one or two individual mechanisms, either ignoring or simulating the other aspects. Since there is a great deal of hand-engineering of various aspects, the line between *origin* of (some aspect of) life research and hand-engineered existing life research is not necessarily clear.

### (e) Physical implementations

The current article focusses on virtual ALife, where it is simpler to implement and study single mechanisms in isolation, potentially giving a clearer picture of their properties and behaviours. But there is also work on ‘bottom up’ physical ALife, briefly overviewed in this subsection.

Biochemical molecules are those that have been produced during the evolution of life, and can be used to support metabolism, autocatalysis and homeostasis, information storage (DNA), interpretation (ribosomes) and activity (RNA and proteins). Wöhler’s 1828 synthesis of the organic compound urea demonstrated that biochemical compounds could be synthesised from non-living materials (although the significance of the work in refuting vitalism may have been overstated [40]). The Miller–Urey experiment [41] demonstrated that amino acids, the building blocks of proteins, could be synthesised from gasses supposed to be in the atmosphere of a primitive earth. Cell membranes that can selectively transport large molecules across themselves, are being designed [42]. Gánti’s extensive Chemoton theory [6,43] focusses on the principles underlying the construction of biological systems: it is a theory of chemical fluid machines, covering self-reproducing chemical cycles, information replication, compartments, evolution, and more. Putting several of these ideas together, protocells [19,44,45] are bio-like minimal cells built from scratch, from the bottom up.

Physical systems can exploit, and are constrained by, the laws of *thermodynamics*; the environment provides a source of free energy to power behaviours, and the constraints prevent runaway reproduction and provide a driver of natural selection. In turn, life accelerates the rate of entropy production [46,47]. Inside a physical being, energy and material obtained from the environment provide the motive power to drive behaviours, and substance to grow bodies, through metabolic processes. Chemobrionics [48] studies the physics and chemistry of self-organizing non-equilibrium systems with semi-permeable membranes.

Large inorganic molecules [49] and complexes [50] have been synthesized in the laboratory, and may provide alternative resources and pathways; automated implementations of search algorithms can be used in their discovery [51]. Inorganic molecules can be assembled into inorganic chemical ‘cells’ [52]. Other inorganic molecules form the basis of experiments in mobile droplets, converting chemical to mechanical energy [53]. Čejková *et al.* [54] review research into inorganic droplets as animated soft matter. Highly engineered meta-materials, with both complex functional and computational properties, may be exploited to provide other sources of complexity.

Robotics research can adopt many ideas from ALife, even if the robots themselves are not usually considered to be alive. Indeed, the idea of self-replicating robots has a long history [55], and there is a thriving current research field on self-assembling robotic systems [56] and self-assembling smart materials incorporating electronics [57]. Sub-branches or robotics with a strong ALife flavour include evolutionary robotics [58] and the embodied intelligence of soft robots [35,59].

## 4. Current virtual ALife research

### (a) Complexity

#### (i) Artificial worlds

Living beings do not exist in a vacuum: they are part of a living system that interacts with and depends on a non-living but complex open-ended environment: they are *embodied*. In a full living system, the environment needs to be engaged in open-ended feedbacks. For example, consider the way life on earth has deeply influenced the planet’s geology, by affecting the water cycle and by enabling the production of new rock types such as limestone.

For physical ALife, its environment might be the natural world or a constrained laboratory subset of it. It naturally experiences thermodynamic resources and constraints, although some of these may also be modulated artificially, such as external provision of food and removal of waste. Many biological experiments similarly artificially constrain the system and environment (e.g. inbred laboratory mice in sterile conditions) such that it is possible that the systems under study are significantly divorced from the systems in the wild: they become a form of ALife.

For virtual life, a complex embodying virtual environment is needed [60]. In many virtual ALife systems, this environment is relatively trivial compared with the complexity of the real world, for example, a two-dimensional spatial grid with scattered resources renewing according to some fixed rule in an agent-based model. In particular, virtual systems tend either to ignore thermodynamic constraints, or implement a simple energy model that imposes artificial constraints with little or no feedback, and no consideration of entropy.

#### (ii) Chemistry

Natural chemistry provides a source of open combinatorial constrained complexity. Artificial chemistries (AChems) [61,62] are used to generate complexity through combinatorial construction. An AChem is a computational system comprising three parts: a set of primitives, analogous to atoms and basic molecules, that underlie the built components; a set of reaction rules that define how the components combine; and an algorithm defining how the components are brought together to react. Particular AChems range from relatively faithful simulations of aspects of natural chemistry, to having highly abstract primitives and rules (such as Fontana’s **λ**-calculus AChem [63]); the underlying purpose is to generate constrained complexity. Symbolic AChems may struggle with intrinsically defining new rules for reacting new kinds of components that arise in the system. Subsymbolic AChems [64,65] have been designed where bonding properties of novel components emerge from structural and dynamic properties of those components.

Automata chemistries are a form of AChem where the ‘atoms’ are assembly language instructions or opcodes, the ‘molecules’ are strings of opcodes forming small programs, and their behaviour is the execution of the program. The assembly language is typically hand-designed to have desired capabilities, such as copying opcodes, and runs in a virtual environment via an interpreter. So AutChem molecules are structurally relatively simple strings, but behaviourally complex program executions. Early work on assembly language ‘organisms’ includes Core Wars [66] and Coreworld [67]. Several AutChems have been well developed over the years, forming small ecosystems of research results. These include Ray’s Tierra [68], Avida [69], Aevol [70], Lerymenko’s minimal Nanopond system [71] and Stringmol [72]. These systems are mostly used to run evolutionary experiments, where imperfect copying results in mutated molecular programs with different execution behaviours.

#### (iii) Compartments

Compartmentalization can provide complexity in several ways [73]. Compartments provide a route to the ‘closed in space’ requirement of autopoiesis, by providing a boundary between self and other. They can support complex behaviours by stopping homogenization: they can support the existence of different compositions and the occurrence of different processes at different spatial locations. And they can support hierarchical complexity through tree-structured nesting of compartments. Some research is in the construction of membranes from smaller components to form compartments [74,75], other in the development and use of mathematical theories of membranes and compartments, such as Păun’s P-systems [76,77] and Cardelli’s Brane calculi [78].

### (b) Autopoiesis and self-* processes

Autopoiesis is here defined as a requirement-level property of life, rather than a design-level process or mechanism, but is often an aspect of specific implementation and study.

As McMullin [30] points out in his review of the history of computational autopoiesis, realization of a minimal model of autopoietic organization in *virtual* systems was part of Varela & Maturana’s initial definition [16], before attempts were made to implement it in *chemical* systems. Indeed, this minimal model does not include physical, energetic or thermodynamic considerations, only abstract organizational ones [17, p. 89]; other authors have built autopoietic models grounded in physico-chemical reality, with important consequences for autonomy and open-ended evolution [79]. The original abstract model has been successfully implemented in a computational system; McMullin [30] notes that although the original implementation included some subtle changes to the original model in order to work [80], that does not invalidate the model, nor does it imply that autopoiesis is uncomputable as some claim [81]. McMullin concludes his discussion by saying that ‘computational autopoiesis continues to provide an effective framework for addressing key open problems in artificial life’.

Alongside studies of fully fledged autopoiesis, there is much work on implementing various self-* processes. For example, Miller [82] successfully developed a cellular program that could construct a representation of the French national flag; the same program could repair the flag if it were damaged. Miller’s cells contained a particular update program, which was discovered through use of an evolutionary search algorithm. An alternative search algorithm is used in Neural Cellular Automata (NCA) [83], where cells contain a particular update neural network. With NCAs, a neural learning algorithm is used to find a neural network that self-produces, and self-repairs, images [84]. These simulations help confirm the notion that a single mechanism can underlie both growth and repair, in a process of homeorhesis. Other forms of self-construction and self-growth are discussed in §4*e*; self-reference and reflection are discussed in §4*f*.

### (c) Agency

The topic of agency tends to be more in the scope of Artificial Intelligence and cognitive science research than that of ALife. For example, agency is often modelled in terms of the belief–desire––intention (BDI) approach [85,86]. Dennett [87] tackles intentionality from a different perspective, that of taking the *intentional stance*: treating a system ‘as if’ it were intentional can simplify descriptions and aid understanding, particularly of engineered systems. If artificial systems can be fruitfully analysed *as if* they were intentional, who is to say when a system is ‘really’ intentional, or what that might mean?

One issue with the AI approach to agency, from an ALife perspective, is that AI typically assumes a relatively complex reasoning agent. ALife, particularly when thinking of origins, is more concerned with much simpler agents, those that are on the edge of fulfilling the requirements for life. This requires a model of agency applicable to these much simpler systems. Levin and Davies [18,88] introduce the concept of agential materials and systems that exist on a *spectrum* of ‘persuadability’, of how much effort is needed to induce them to a particular behaviour, ranging from full low-level control (little agency) to just high-level instruction (full agency). Agency is not all or nothing: more persuadable systems have more agency, more ability to perform autonomously.

### (d) Adaptation

The main adaptive process investigated in ALife experiments is neo-Darwinian evolution, with a genome encoding information and a reproductive process either hard-coded, or emergent. Other adaptive processes are used at the individual, rather than population, level; these include epigenetic processes, neural learning and artificial immune systems.

#### (i) Evolution

In evolution, *replication* is the process of making a copy (a replica) of the genetic information, whereas *reproduction* is the process of making a new offspring from a parent or parents. In many evolutionary experiments, replication of the genetic material with mutation, and reproduction of the organism, are hard-coded as ‘short-cuts’. In some cases, the process of replication or reproduction is the focus of the experiment.

A symbolic Artificial Chemistry (AChem; see §4*a*(ii)) can be defined with rules specifically designed to enable reproduction: a set of molecules follows the rules of the AChem to produce another similar set, possibly with some degree of mutation [75]. Cell division may be modelled explicitly using membrane systems. Automata Chemistries (AutChems) are often used to investigate processes of replication with mutation, with the dynamics of the executing program being the analogue of the dynamics of the replication process. AutChem replication may be examined from a starting point of hand-crafted replicating programs (as is typical with Tierra, Avida, Stringmol), or from scratch, allowing replicators to emerge (Nanopond, and even conventional programming languages [89]). Another distinguishing feature is whether the evolving systems are driven by an externally supplied fitness such as performing a task (artificial selection), or an internally driven survival process (natural selection).

von Neumann’s pioneering work on designing a universal replicating system in a cellular automaton (CA) [90] foreshadowed the architecture of replication in biological systems: an information-bearing ‘tape’ that is, at different stages of the process, interpreted as instructions for copying, and itself copied. Like many CA systems, it is highly fragile to mutation, and so such an implementation is not a suitable substrate for mutation experiments. Experiments using the same abstract architecture in different automata chemistry substrates have differing successes: an Avida implementation tends to degenerate to a self-copier [91]; a Tierra implementation is not mutationally robust [92]; a Stringmol implementation finds several instances of semantic closure, where different parts of the architecture can mutate successfully [29]. Stringmol uses a model of replication originally discussed by Neumann & Burks [90], of requiring one string to copy another, rather than of self copying. It is also notably ‘softer’ than other AutChems [93], indicating that such softness may be needed for a successfully mutating system.

#### (ii) Learning

As noted in §3*c*, Darwinian evolution is the ground floor of Dennett’s Tower of Generate-and-Test, and more complex beings have access to higher-level adaptation and learning mechanisms.

Transitions between different levels of neural learning can be identified [94]. Reinforcement learning [95], which models the next, ‘Skinnerian’, floor in the Tower, is a popular approach to neural learning through environmental feedback. Local learning algorithms are being developed for physical neural networks to overcome communication issues inherent in implementing the back-propagation algorithm; these also have advantages in digital systems of better parallelizability and reduced memory usage [96,97].

Not all higher-level adaptation need take place through neuro-cognitive learning. The adaptive immune system has memory and learns new responses to invasive events [98]; algorithms based on various immune system processes abound [99]. A network model of ecosystem interaction strengths, with a Hebbian learning approach to adjust those strengths, can be used to model adaptation at the ecosystem level [100,101]. A body can adapt to environmental changes, from homoeostatic processes to adaptive growth (see §4*e*).

#### (iii) Meta-adaptation

Adaptation applies to the structure and behaviours of the adapting system, and also to the process of adaptation itself. The adaptation of adaptation, or second-order adaptation, is necessary in living systems because they are embodied in open complex environments that provide resources for self-construction, constraints that supply adaptive pressures and engage in feedbacks that cause changes to the environment too. As adaptations themselves adapt to become more effective, the adapting systems will preferentially thrive, and learn to adapt to more challenging conditions.

Meta-learning, or learning how to learn, started in the education domain, discussing the self-reflective nature of the topic: being able to examine and change one’s own state and behaviours. This entered the digital domain through the idea of reflective programming [102]: programs that can examine and change their own code. Such reflection does not need to be a conscious process; for example, programs could change their own code through an evolutionary process, such as the Automata Chemistries discussed in §4*d*(i) [103]. Meta-learning is related to the study of the evolution of evolvability and of evolution itself [104,105]. Some argue that evolution can exploit a higher-order learning process [106]. Meta-learning can be aided by the need to solve progressively more complex problems, requiring progressively more sophisticated techniques. Co-evolution of learning and problem difficulty [107] is potentially a route to open-ended systems (see §4*f*).

### (e) Morphogenesis

#### (i) Growth processes

Growth processes take a compound individual from an initial single cell-like state into a mature adult. Morphogenesis is differential growth resulting in shape and structure. Doursat *et al.* [108] provide a collection of different approaches to building morphogenetic systems.

L-Systems, a form of generative grammar, or rewriting grammar, are symbolic systems initially invented to model the growth of multicellular organisms [109]. Originally deterministic parallel rewriting systems, these have been extended to include context, probabilistic rule application, environmental input [110], internal chemical flows [111] and more, as more sophisticated mechanisms were required to model realistic plants. Generative grammars have been used to ‘grow’ more types of things, such as tables [112], architectural shape [113], robots [114] and body morphologies [115]. An alternative, agent-based approach to morphogenesis is through a cellular-like division process, producing tissue-like structures, or neural network structures [116,117]. Components divide, differentiate and move in a network topology or in two-dimensional or three-dimensional space.

As an alternative to a single ‘seed’ growing through rewriting or division, a self-construction process takes individual components and assembles them into a whole. DNA nanotechnology [118] exploits the informational content of matter. DNA tiling programmatically ‘glues’ small patches of material into an array of a larger structured whole; theoretical models have been experimentally implemented [119,120]. DNA origami uses DNA ‘staples’ to fasten a long single-stranded DNA into preprogrammed shapes [121,122].

#### (ii) Evo-devo

So-called evo-devo systems combine the processes of *evolution* of an information-bearing genome and *development* from that information to an adult phenotype subject to the selection pressures. Bateson [123] distinguishes evolutionary and other learning processes (see §4*d*) that require some randomness to feed creativity and exploration and to generate new information, from more deterministic ‘becoming’, or growth, processes that can be pure iterative unfolding from a previous state. In practice, growth also includes some randomness owing to interaction with a varying environment, yet early stages of growth, at least, may be constrained to a more controlled environment (the womb, a nest, a school).

Generative encodings have been found to aid the evolution of structures with symmetries [112,124] and are often used as the developmental stage in evo-devo algorithms [114,115]. For systems with behaviours, such as robots, the evolutionary step often co-evolves instructions to grow both the body and an embodied controlling neural network [125,126]; an additional learning process during development may also be included [58,127].

Early virtual experiments mostly separate the evolution of the genome from the subsequent application of given growth rules. Evolution of the developmental process itself often makes use of growth rules encoded as an evolving neural network [83,117], using neuro-evolution techniques. Evolution of growth rules is a step on the route to open-ended evolution (§4*f*).

### (f) Open-endedness

One of the requirements for life stated in §3*a* is that of *open-ended* (OE) adaptation. This is a topic of great interest in ALife [128–131]. Definitions are hard to come by, however. Typically, definitions reduce to ‘displaying continual novelty’, but do not further define ‘novelty’.

Banzhaf *et al.* [132] provide a definition and they identify three levels of novelty with respect to a model and metamodel of the system in question: *variation* is novelty within a model, different instances in the model; *innovation* is a novelty that changes the model, with a new or changed type, relationship or behaviour not present in the current model; *emergence* (later renamed *transformation* [133]) is a novelty that changes the metamodel, with a new or changed concept, a new kind of type or behaviour not present in the current metamodel. Then an OE system is defined to be a system with the ability to continually produce innovations and transformations. According to this definition, continual variation alone is not sufficient for a system to be called OE: the system is merely exploring a pre-determined space; innovation and transformation change the space being explored.

Open-endedness is extremely hard to achieve under this definition: an innovation or transformation grows the search space, but exploration of that new search space is then mere variation; further innovations or transformations are needed to continually expand the possibilities. This requirement of continually moving outside the model, of lacking a pre-determined search space, has a consequence: many kinds of simulations, such as agent-based models that have a pre-determined space defined by their fixed code, cannot exhibit OE behaviours. Self-modifying code that can change the underlying model, possibly achieved through direct overwriting of existing code as in Automata Chemistries (see §4*d*(i)), or through higher-level self-referential computational reflection processes [102,134], would be needed. Another consequence is that measures and tools for analysing OE systems need themselves to be OE in order to track the model changes [135]. Further, any fitness or other pressure on the system’s adaptation should itself be open to changes in the system, not some closed constant value, or else adaptation will converge. Additionally, combinatorial complexity alone may not be enough: a system may need to contain increasing information to be OE [136].

## 5. Conclusion

The ability of virtual life experiments to isolate single mechanisms to study is both a strength and a weakness. By using programming ‘shortcuts’ [132] to hard-code critical supporting mechanisms not under study, and by simply ignoring inessential mechanisms, virtual ALife experiments can have a precise focus on the mechanisms of interest. This property may even be used to quantify ‘how alive’ a virtual system is: which requirements (§3*a*) are met by the endogenous mechanisms, which are provided externally by shortcutting, and which are not present at all? Furthermore, in a digital system, it is possible to log and document low-level variables with relative ease, allowing sophisticated analyses of the processes under study. Where the experiments concern virtual ALife, rather than simulations of physical ALife, the ‘reality gap’ [137] is not relevant: there is no gap. Shortcutting, or ignoring, mechanisms is more problematic in physical ALife experiments, as is measuring the variables of interest. However, this strength becomes a weakness when trying to combine mechanisms to form full beings or systems because there is typically little coherence between the separate mechanisms so studied. Differing virtual substrates, assumptions and interfaces make it highly unlikely that the various individual parts can be composed into more complex and complete wholes. There is no overall framework for such composition. Physical ALife components at least share an underlying physical reality. Additionally, virtual worlds tend to be two-dimensional, both to reduce computational load and to aid visualization. Three-dimensional topologies are more powerful: for example, channels can cross without interfering. In a virtual world, higher-dimensional environments.^7^ or other exotic topologies, are available, but are more difficult to visualize and comprehend.

Here, we suggest some steps that could improve the likelihood of virtual ALife being originated.

### (a) Requirements

— *Autopoiesis*. Many of the mechanisms used to study or implement autopoiesis and self-* mechanisms are symbolic and fragile, and so are difficult to integrate with evolutionary processes. ‘Softer’ sub-symbolic substrates that use evolution or learning to search for the mechanism [29,64,65,82–84,93] may provide a route to developing more robust processes that can be combined.
— *Self/agency*. Work on the autonomy of simple systems is needed, especially how one might exploit the autonomy of a variety of functional parts to construct more complex wholes. Ideas of agential material are relevant here [18,88].
— *Open-ended adaptation*. Living beings are embodied in a complex co-evolving environment that provides resources and poses challenges, yet many virtual ALife systems exist at best in a relatively simple world, at worst in a virtual vacuum. Open-endedness also requires self-reference and self-modification at a deep level, to permit the innovation and transformation of the processes [132,133].

### (b) Design framework

Operationalizing all the requirements would be aided by a common design framework, into which different components could be designed, implemented and integrated. Such a framework would need to consider the following issues.

— Designs for parts to be composed into whole organisms. Since life is processual, not merely structural, the design should support composition of processes.
— Designs for individuals interacting with other individuals. Interaction requires sensing and actuating, implying the need for communication protocols and mutual changes and effects.
— Designs for complex, co-evolving non-living environments engaged in mutual feedback with embodied living individuals. Designs should consider some analogue of an energy/entropy model, to provide resources and constraints.
— Designs for change, in individuals, populations and environments, that support soft, open-ended processes that can introduce innovations and transformations in the system.

### (c) Implementation issues

Such a design framework need not be overly restrictive, as it could support multiple different instantiations—different artificial worlds—with different implementation choices. Each artificial world would then need to define specifics for its components, composition, interaction and change. Hence it is crucial for a given world to include documentation of its structures and interfaces, and for given components also to document their assumptions and capabilities. This would allow a library of components for a given world to be built up and used by different researchers. It could also include a library for composition, for example, of specially designed ‘agents’ used to translate between different formats and protocols of different components.

Much progress has been made in the domain of virtual ALife. This is currently mainly limited to single mechanisms, however, with the goal of a deep understanding of those mechanisms. Even such results may be considered to be partially alive, to the extent that they meet some, but not all, of the requirements of life. To achieve the goal of a complete living virtual being, or living virtual system, requires building on this success, to combine and integrate such mechanisms, and to acquire a deep understanding of the effects of such composition and interaction.

## Data Availability

This article has no additional data.

## Footnotes

^1^
For completeness, one should include the possibility of *alien* artifice, but given that there is as yet no compelling evidence of extraterrestrial *natural* life, distinguishing extraterrestrial *artificial* life would be challenging. Discovery of alien von Neumann probes [7] would be one compelling piece of evidence.

^2^
Varela *et al*. [16] state: ‘The autopoietic organization is defined as a unity by a network of productions of components which (i) participate recursively in the same network of productions of components which produced these components, and (ii) realize the network of productions as a unity in the space in which the components exist. Consider for example the case of a cell: it is a network of chemical reactions which produce molecules such that (i) through their interactions generate and participate recursively in the same network of reactions which produced them, and (ii) realize the cell as a material unity. Thus the cell as a physical unity, topographically and operationally separable from the background, remains as such only insofar as this organization is continuously realized under permanent turnover of matter, regardless of its changes in form and specificity of its constitutive chemical reactions’.

^3^
Of course, were the simulation hypothesis [20] true, the question would be moot.

^4^
Aristotle's *efficient cause* is that which brings a thing into being, or initiates a change in it. The causal agent can be internal to the thing, which is what Rosen requires for a living organism, or external, such as the thing's manufacturer.

^5^
Living systems are *thermodynamically open* (to a flux of material, energy and waste) but *organizationally closed* (the organized components of the self act to produce the organized components of the self).

^6^
The unstructured random search generation algorithm may be relegated to the Tower's basement.

^7^
A Greg Egan short story [138] has a sixteen-dimensional virtual world implemented by three-dimensional physical Wang tiles; similarly imaginative virtual ALife worlds could be investigated.
